# Best Left to the Experts: Proficiency and Experience are Key for Safety in Remote-Access Thyroidectomy for Graves’ Disease

**DOI:** 10.1007/s00268-022-06496-y

**Published:** 2022-03-01

**Authors:** Claire E. Graves, Michael J. Campbell

**Affiliations:** Section of Endocrine Surgery, Department of Surgery, University of California Davis, Sacramento, CA USA

Graves’ disease (GD) is the most common cause of persistent hyperthyroidism in adults. Treatment options include antithyroid drugs, radioactive iodine (RAI), and surgery. Despite being an effective and definitive treatment [1], surgery is the least commonly employed therapy in the USA. The etiology of this low utilization of thyroidectomy is likely multifactorial, but in some patients, concern regarding the cosmetic effects from a visible cervical scar may play a role. To address this issue, a variety of remote-access thyroidectomy approaches have been developed in recent years with the goal of eliminating the traditional cervical scar–either by moving incisions to more “hidden” areas (e.g., axilla, breast, or posterior auricle) or by accessing the thyroid through the oral vestibule.

In this issue of *World Journal of Surgery*, Bu Bshait et al. report their series of 51 patients with GD who underwent robotic transaxillary thyroidectomy (RTT) and 141 GD patients who underwent thyroidectomy via traditional cervical approach [2]. All robotic procedures were performed successfully without conversion to open. The authors report an overall complication rate of 33.3% in the RTT group vs. 22.7% in the open cohort. This difference was not statistically significant (*p* = 0.1), though statistical analysis was limited due to patient numbers. In both groups, the predominant complication was transient hypocalcemia (21.6% RTT, 17.7% open, *p* = 0.3) with more clinically severe complications such as hematoma (2.0% RTT vs. 1.4% open, *p* = 1.0), recurrent laryngeal nerve injury (2.0% RTT vs. 0% open, *p* = 0.3), tracheal injury (2.0% RTT vs. 0% open, *p* = 0.3), and permanent hypocalcemia (2.0% RTT vs. 2.1% open, *p* = 1.0) being infrequent and similar between groups. The transaxillary group did suffer from one transient brachial plexus injury and one skin flap burn, unique to the transaxillary access approach.

Importantly, these results should be interpreted considering several points of context. First, the robotic cohort in this study represents a highly selected group. The authors note that, among other considerations, patients in the robotic group were initially selected for “relatively normal” thyroid volume. Though this restriction was lifted during the study period, the mean gland weight was significantly lower in the robotic cohort compared to open (mean 89.4 g vs. 116.7 g, *p* = 0.03). The smaller thyroid volumes likely contributed to improved visualization and retraction in the RTT group and may limit the generalizability of these data. Second, patients underwent subtotal thyroidectomy, with approximately 3 g of thyroid tissue remaining in the thyroid bed. In the USA, total thyroidectomy is typically performed for GD due to lower recurrence rates, but total thyroidectomy is associated with increased risk of postoperative hypocalcemia compared to subtotal resection [3].

Finally, the importance of practitioner and institutional expertise with this specialized technique cannot be overstated. The authors initiated the transaxillary endoscopic technique at their institution over twenty years ago in 2001 and introduced robotic technique in 2007. They have previously reported their experience with over 5000 cases of robotic thyroidectomy, with GD comprising only a small subset of their overall cohort [4]. They note that approximately 75% of the patients in their robotic group had surgery after at least 5 years of experience with RTT. GD, with its known increased risks of hematoma, bleeding, and hypocalcemia when using an open cervical approach, has traditionally been considered a contraindication to remote-access approaches [5]. However, with increasing practitioner experience and comfort with this technique, the authors expanded its application.

Remote-access thyroidectomy techniques provide the additional benefit of a superior cosmetic result, which can factor into patient and provider decisions about the treatment of GD. The authors provide promising data that RTT can be safely performed for GD, but it is important to note that they are uniquely suited to perform robotic thyroidectomy in this higher risk patient population. Surgeons must be cognizant that similar excellent outcomes can only be achieved with careful, stepwise implementation of RTT and that surgeon and institutional experience are key to minimizing complications in remote-access thyroidectomy for GD.
